# Autonomic nervous system activity changes in patients with hypertension and overweight: role and therapeutic implications

**DOI:** 10.1186/s12933-021-01356-w

**Published:** 2021-08-19

**Authors:** Paul Valensi

**Affiliations:** Unit of Endocrinology, Diabetology and Nutrition, Jean Verdier Hospital, CINFO, CRNH-IdF, AP-HP, Paris Nord University, Avenue du 14 Juillet, 93140 Bondy, France

**Keywords:** Autonomic nervous system, Hypertension, Obesity, Type 2 diabetes, Selective imidazoline receptor agonists

## Abstract

The incidence and prevalence of hypertension is increasing worldwide, with approximately 1.13 billion of people currently affected by the disease, often in association with other diseases such as diabetes mellitus, chronic kidney disease, dyslipidemia/hypercholesterolemia, and obesity. The autonomic nervous system has been implicated in the pathophysiology of hypertension, and treatments targeting the sympathetic nervous system (SNS), a key component of the autonomic nervous system, have been developed; however, current recommendations provide little guidance on their use. This review discusses the etiology of hypertension, and more specifically the role of the SNS in the pathophysiology of hypertension and its associated disorders. In addition, the effects of current antihypertensive management strategies, including pharmacotherapies, on the SNS are examined, with a focus on imidazoline receptor agonists.

## Introduction

Hypertension is one of the leading causes of premature death worldwide with 1.13 billion people having hypertension. It is associated with an increased risk of cardiovascular diseases (CVD; e.g., stroke, angina, myocardial infarction, heart failure, peripheral artery disease, and abdominal aortic aneurysm) as well as end-stage renal disease [1, 2]. Hypertension often co-occurs with other CVD risk factors such as diabetes mellitus, dyslipidemia/hypercholesterolemia, obesity and chronic kidney disease [1, 3, 4]. Despite several actions set up to improve diagnosis, management and awareness about hypertension, the incidence and prevalence are still increasing [5, 6]. The prevalence of hypertension is higher in low- and middle-income countries [5] and increases with age [1].

The autonomic nervous system has been implicated in the pathophysiology of hypertension [7, 8] and treatments targeting the sympathetic nervous system (SNS) have been developed [9, 10] although largely forgotten or ruled out in international recommendations [1, 2]. The aim of this review, therefore, is to examine the pathophysiology of hypertension, in particular the role of the autonomic nervous system (ANS) and medications that target the SNS to help control hypertension. A search of PubMed was conducted using search terms such as “hypertension”, “blood pressure”, “sympathetic nervous system” and “SNS”, with no restrictions on date of publication. Supplemental, focussed ad-hoc searching was conducted where necessary.

## Hypertension

According to the ESC/ESH guidelines, hypertension is defined as office systemic blood pressure values ≥ 140 mmHg and/or diastolic blood pressure values ≥ 90 mmHg [2].

The aetiology of hypertension is currently still poorly known, although several risk factors have been identified for its development such as overweight, diet (e.g., sodium intake), physical activity, and alcohol consumption [11, 12]. Its pathogenesis is multifactorial and highly complex, involving multiple organ systems and numerous independent and interdependent pathways [13]. Known systems involved in blood pressure control include cardiovascular, renal, neural, and endocrine systems, as well as local tissues, with the kidneys playing a central role [13]. In addition, genetic factors, and activation of neurohormonal systems are involved in the pathogenesis of hypertension. The neurohormonal system is responsible for maintenance of cardiovascular homeostasis, with the sympathetic nervous system (SNS) and the renin–angiotensin–aldosterone system (RAAS) being two key components [14]. RAAS is involved in the maintenance of arterial blood pressure, plasma sodium concentration and extracellular volume, and needed for the function of the heart and kidneys. RAAS dysfunction can lead to chronic diseases development as hypertension or heart failure [15, 16].

While animal studies have demonstrated extensive and reciprocal interactions between SNS and RAAS important to cardiovascular regulation and the development of hypertension, supportive evidence in humans is currently lacking [17, 18].

## The sympathetic nervous system

### Physiological role of the sympathetic nervous system

**Fig. 1:**
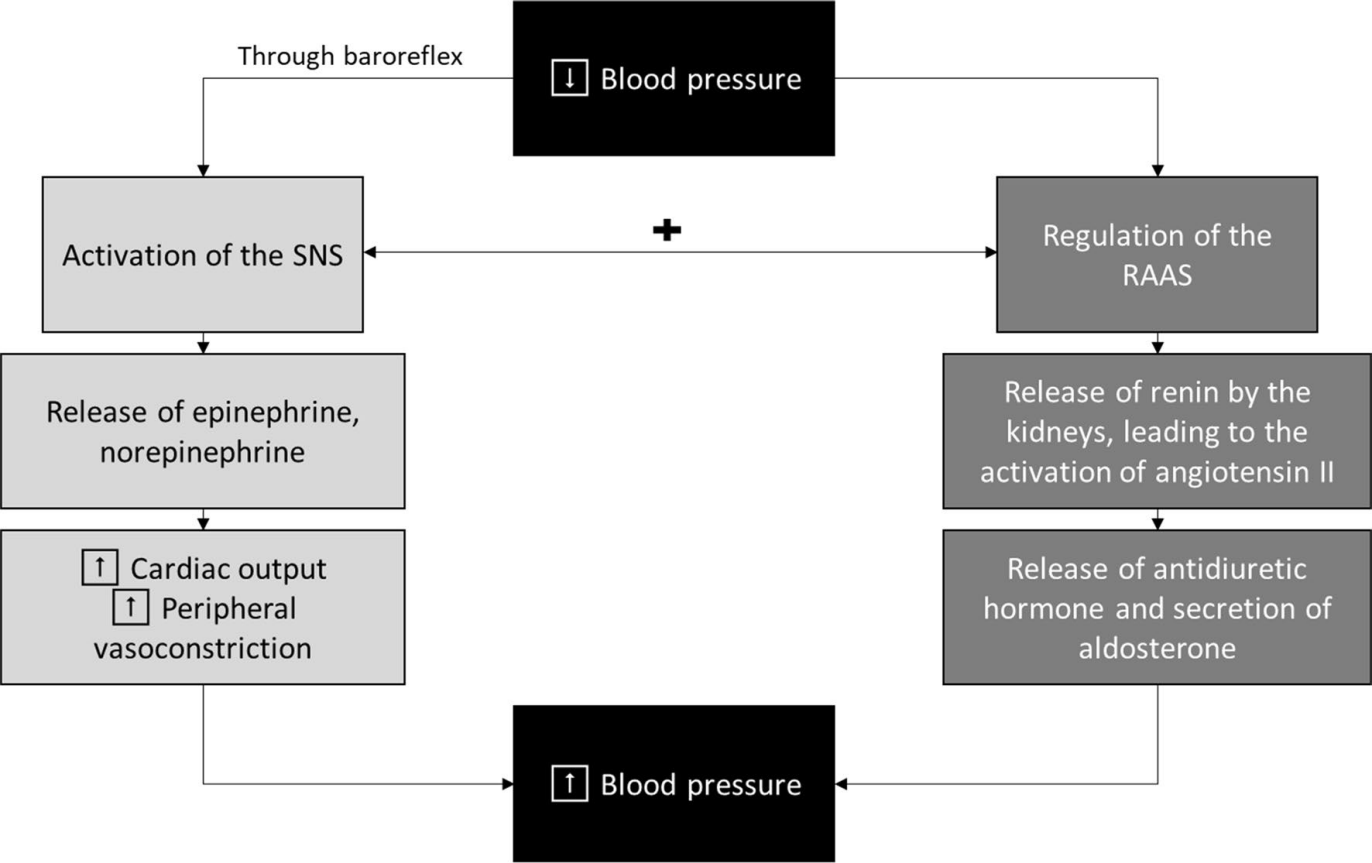
Control of blood pressure by the sympathetic nervous system (SNS) and the renin–angiotensin–aldosterone system (RAAS)

Maintenance of cardiovascular homeostasis requires continual redirection of blood flow to ensure adequate blood supply to active tissues. Under normal functioning, the autonomic nervous system, comprising the sympathetic, parasympathetic and enteric nervous systems, makes unconscious adjustments in regional blood flow and cardiac output, and coordinates with the central respiratory network in order to respond to varying metabolic and thermoregulatory demands [19]. The SNS is activated when baroreceptors, specialised stretch receptors located within thin areas of blood vessels and heart chambers, sense changes in pressure [20]. When arterial pressure drops, the SNS is immediately activated resulting in increased cardiac output and vasoconstriction of peripheral vessels (Fig. 1) [20]. Subsequent constriction of renal afferent arterioles results in activation of the renin–angiotensin–aldosterone system and secretion of renin [14].

The SNS plays an important role in the regulation of liver metabolic processes, with the sympathetic and parasympathetic systems working in tandem to respectively stimulate and suppress hepatic gluconeogenesis, and insulin stimulating glycolysis and lipogenesis, and suppressing gluconeogenesis [21]. Overall, the SNS enhances the production of glucose by the liver and mobilises metabolic fuels for use by the tissues [21]. In the pancreas, Langerhans islets are innervated by autonomic nervous system fibres resulting in modulation of insulin secretion with parasympathetic nerves stimulating insulin secretion and sympathetic nerves having the opposite effect [22, 23]. Enhanced lipolysis in adipose tissue by the SNS is part of a control mechanism designed to break down fats [24].

### SNS overdrive in hypertension

**Fig. 2:**
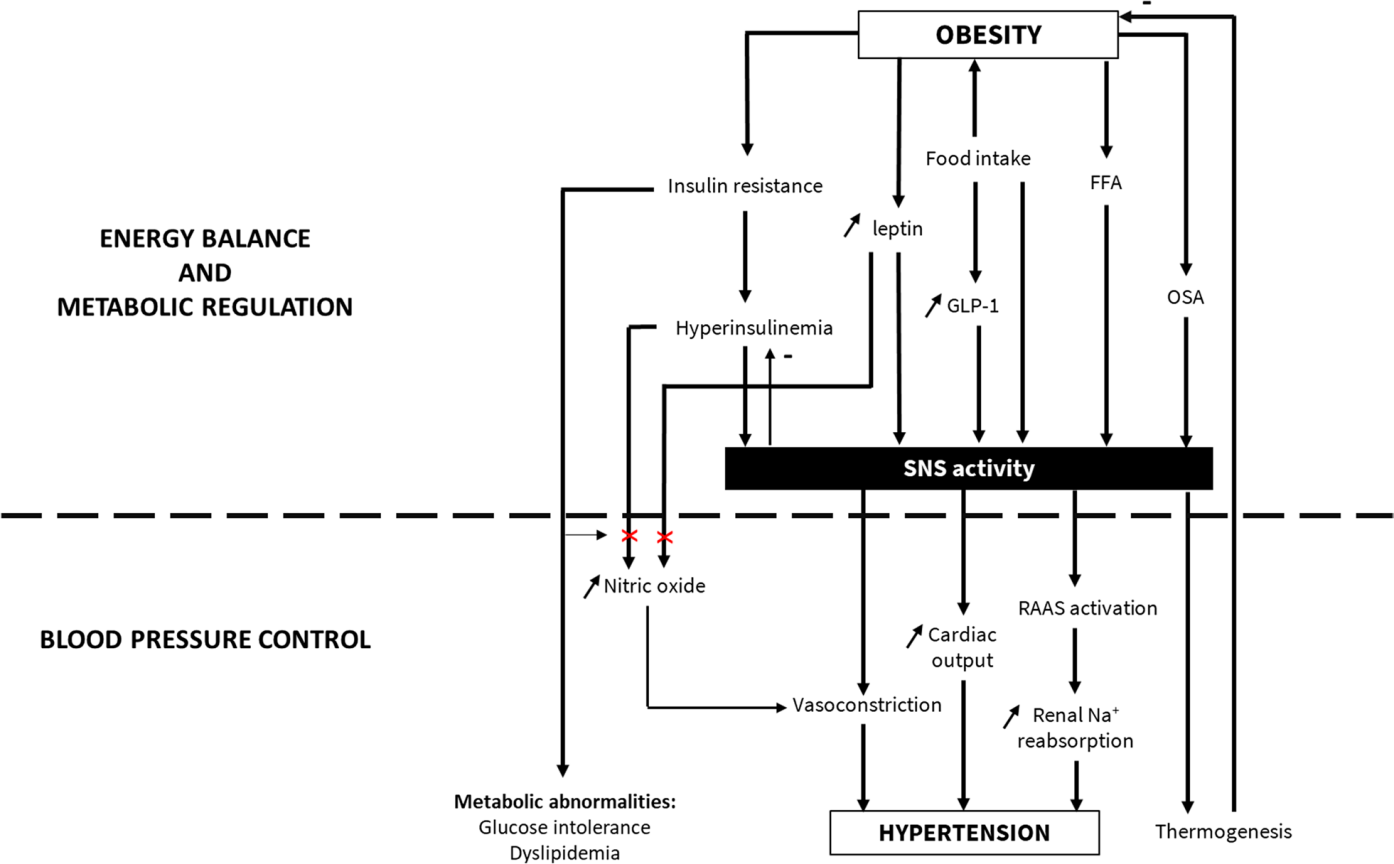
The role of the sympathetic nervous system (SNS) in energy balance and metabolic regulation, and in blood pressure control in overweight patients. *FFA* Free fatty acids, *OSA* obstructive sleep apnea, *RAAS* renin–angiotensin–aldosterone. Red crosses designate a disruption

The consequences of sympathetic overdrive leading to hypertension are numerous and include cardiovascular, renal and metabolic effects (Fig. 2) [25–27]. A state of sympathetic activation is associated with increased heart rate, and appears to promote cardiac and vascular alterations [25, 28], contributing to the development of major complications of hypertension such as arrhythmia, left ventricular hypertrophy and increased arterial stiffening [25–29]. The SNS also appears to affect haemostasis, with acute activation of the SNS resulting in hypercoagulability due to increased platelet aggregability [29, 30].

Sympathetic activity is increased in hypertension and heart failure, and is responsible for initiation and development of the diseases [7, 8, 31]. While specific causes of this increase are mostly unknown, genetic influences, behavioural and lifestyle factors appear to be involved [7, 8]. Increased SNS activity is believed to contribute to the pathophysiology of heart failure through multiple mechanisms, including desensitization of cardiac β-adrenergic receptors, adverse effects on excitation–contraction coupling, and fibrosis [16, 32].

The effects of the SNS activation are mediated by adrenergic neurotransmitters (norepinephrine, epinephrine and dopamine) having vasoconstriction properties [18]. It has been shown that the norepinephrine spillover is increased in patients with high blood pressure, and this increase is mainly seen in the heart and the kidneys, both tightly involved in blood pressure control [18]. The release of these adrenergic neurotransmitters induces action on cardiovascular and metabolic systems by targeting organs involved in homeostasis control as the heart, kidneys, veins, and arterioles, and leading to renin release, sodium retention, increase in heart rate, arrhythmias, and left ventricular hypertrophy. Thus, these targets of the SNS contribute to increase blood pressure. Regarding the metabolic effects, the adrenergic neurotransmitters are responsible for action on different organs and tissues: on fat cell to increase lipolysis resulting in an increase in fatty acid release, on the liver to increase gluconeogenesis, on pancreatic β-cells to decrease insulin secretion. Conversely, free fatty acids may enhance sympathetic activity [33]. If the sympathetic activity is raised chronically, it may lead to development of insulin resistance and hypertension, increasing the risk of cardiovascular diseases. The role of therapeutic inhibition of sympathetic overdrive in the prevention of the metabolic disorders and the associated adverse outcomes requires adequate testing in properly sized randomised controlled trials [8, 21, 24, 31, 34–38].

In contrast to increased sympathetic activity, parasympathetic activity is decreased in patients with hypertension, suggesting a sympathetic/parasympathetic imbalance [39–41].

A higher prevalence of hypertension is found in diabetic patients with a defective parasympathetic control. Moreover, there is an association between this defect and hypertension profile and vascular complications [42].

As well as being involved in hypertension, abnormal activation of the SNS has been implicated in metabolic syndrome, known risk factors for hypertension [4, 43].

### Obesity, obesity-related hypertension and SNS overdrive

Hypertension is common in obese individuals. It is estimated that about 75% of hypertension incidence is directly linked to the presence of obesity (defined by a body mass index ≥ 30 kg/m^2^).

A dysfunction of the SNS activity leads to weight gain. Indeed the SNS plays a critical role in controlling energy expenditure in response to certain physiological stimuli (e.g., changing energy states, food intake, carbohydrate consumption, hyperinsulinemia, and exposure to cold) via regulation of the resting metabolic rate and initiation of thermogenesis by action on the brown adipose tissue [43, 44].

However, obesity is associated with SNS overdrive which is considered as a compensatory mechanism for the increase in energy expenditure, thus allowing to restore the energetic balance [45]. In particular SNS activity was shown to correlate with visceral adiposity [46]. Such SNS overdrive may contribute to metabolic disorders as insulin resistance with subsequent hyperinsulinemia, impaired glucose metabolism, diabetes, dyslipidemia [47, 48]. Obesity is also often linked to resistant hypertension, characterized by an incapacity to control blood pressure despite the prescription of at least three anti-hypertensives agents, including a diuretic [48].

However, some data suggest that sympathetic response to various stimuli may be blunted in obese patients compared to lean individuals. For instance, sympathetic activity was reported in obese patients to be less stimulated after a meal rich in carbohydrates or oral glucose intake despite a higher insulin response [49–51], and they exhibit a lower hemodynamic response during isometric or heterometric exercise [52], and a lower vasoconstrictive response to sympathetic activation during breathing-in [53]. Such a blunted sympathetic response or sympathetic reserve may promote weight gain and aggravate insulin resistance and subsequent hyperinsulinemia, and thus maintain the vicious circle of weight gain—insulin resistance—sympathetic activation with blunted response—weight gain, etc. Increased leptin secretion by the adipocytes in patients with obesity contributes to vascular and systemic insulin resistance and SNS dysfunction [3, 4]. In addition to being an important regulator of fat accumulation, food intake, neuroendocrine outflow and metabolism, leptin, an adipocyte-derived hormone, plays a role in the development of hypertension by increasing SNS activity in tissues involved in cardiovascular regulation such as the kidneys and blood vessels [54, 55]. Leptin has a key role in energy expenditure regulation through SNS, as it targets the arcuate nucleus to activate the concerned signalling pathways [47]. Impaired adiponectin secretion in patients with obesity is also thought to be involved in SNS activation and the promotion of insulin resistance [4]. Also, insulin-induced inhibition of lipolysis has been reported in obese patients with type 2 diabetes following modulation of SNS activity [37]. Results of animal and human studies suggest that central sympathetic overactivity is involved in the aetiology and complications of metabolic syndrome and its associated components (i.e., abdominal obesity, insulin resistance, hyperglycaemia, dyslipidemia, hypertension, systemic inflammation) [43].

Increased urinary noradrenaline and metabolite levels, and elevated plasma noradrenaline spill over, have been demonstrated in obese adults compared with lean individuals [56, 57]. Also, obese adults display increased resting sympathetic nerve activity in skeletal muscle [58–60]. Obesity is also associated with increased SNS activity to various tissues, in particular the kidneys, leading to increased vasoconstriction and fluid retention, and activation of the renin–angiotensin–aldosterone system, promoting increase in arterial blood pressure [55]. It is believed that these changes are mediated by elevated serum leptin levels due to increased fat mass combined with selective leptin resistance [55]. There is evidence that a reduction in body weight, induced by lifestyle changes, can improve lipid profile, glucose metabolism, insulin sensitivity, and result in lowering systolic and diastolic blood pressure [61]. Some specialised diets have also been shown to reduce sympathetic activity, and improve baroreflex and insulin sensitivity [61]. In addition, both SNS and parasympathetic nervous system (PNS) activities are improved when dietary measures are combined with exercise [62]. Nevertheless, for patients with obesity-related hypertension (OHT) dietary measures are not sufficient to control blood pressure, and pharmacological strategies must be initiated [48].

OHT is often associated with diabetes mellitus, metabolic syndrome and dyslipidemia. Sympathetic overdrive in patients with OHT is often enhanced in those with a sleep apnea syndrome. Leptin, insulin and RAAS may contribute to increase SNS activity. Indeed, clamp studies in healthy and obese individuals have shown that hyperinsulinemia may activate the SNS and reduce PNS activity [63, 64], and so may promote OHT, and that hyperinsulinemia and inappropriate SNS activation contributes to increased renal sodium reabsorption [4]. Besides, in case of insulin resistance, a vascular resistance to the vasodilative effect of insulin is also observed promoting development of hypertension [64]. Similarly, endothelial dysfunction associated with obesity may impair the vasodilative effect of leptin and also contribute to hypertension [65] (Fig. 2).

In addition to sympathetic alterations, a cardiac vagal defect may be detected in more than one third of patients with metabolically healthy obesity [66], suggesting that vago-sympathetic imbalance is an early disorder in obesity which may play a role in subsequent hypertension. Indeed, rats with lesions of ventromedial hypothalamus do not exhibit hypertension despite massive obesity, increased plasma catecholamines and normal heart beta-adrenoceptivity. In this model, increased heart vagal tone was suggested to be protective against the development of hypertension [67].

### GLP-1 and SNS overdrive

The SNS has also been implicated in the control of heart rate via glucagon-like peptide 1 (GLP-1), an incretin hormone released by the gut in response to food intake, which has receptors in peripheral tissues and central nervous system [47, 68, 69]. It is known that GLP-1 has beneficial effects on metabolic parameters in patients with type 2 diabetes such as increase in insulin release, replenishment of insulin stores, pancreatic β-cells proliferation, improvement of insulin sensitivity in skeletal muscle, inhibition of glucagon secretion, decrease in liver gluconeogenesis, decrease in gastric emptying, increase in thermogenesis, reduction of appetite and body weight [70, 71]. As well as glycemic control, GLP-1 plays a role in heart rate control [72, 73], with animal studies suggesting that this GLP-1-mediated heart rate control is associated with sympathetic activation and/or depression of parasympathetic modulation [74–76] and that GLP1-receptor agonists induce an increase in heart rate [69]. In addition, animal studies suggest that GLP-1-enhances sympathetic activity and increases thermogenesis of brown adipose tissue [70]. However, a slight blood pressure decrease is observed during treatment by GLP1-receptor agonists, as a result of weight loss and possibly due other mechanisms including an improvement of endothelial function [77]*.*

### Sleep apnea and SNS overdrive

It has been suggested that activation of the autonomic system caused by baroreflex dysfunction, predisposes to hypertension in people with sleep apnoea, with more markedly impaired baroreceptor reflex sensitivity being significantly associated with increased blood pressure and hypertension in generally healthy elderly individuals with sleep apnea [78]. It has been postulated that the cardio-protective effects of parasympathetic activity are reduced by acute sympathetic activation related to the induction of a downwards resetting of baroreceptor by sleep apnoea [78]. Impaired baroreceptor reflexes and sympathetic hyperactivity have also been observed in heart failure, which is also closely associated with sleep apnea [79].

The increased sympathetic activity is seen in patient with sleep apnea even when they are awake, and the apneic episodes are associated with further increases in sympathetic nerve activity and blood pressure during sleep [80].

It has been shown that an increase in SNS activity is also responsible for an increase in insulin resistance and central adiposity, which are associated with higher rates of sleep apnea [81]. In obese patients with resistant hypertension and sleep apnea, a decrease in sympathetic overdrive leads to a significant weight loss and decrease in blood pressure [81]. Thus, it is justified to screen for sleep apnea in diabetic patients with resistant hypertension, even in absence of symptoms to optimize blood pressure reduction [82].

## Evaluation of SNS activity

Several methods are available to measure SNS hyperactivity including sympathetic nerve recording, radiotracer-derived measurements of regional sympathetic neuronal activity or heart rate and blood pressure variability.

### Microneurography

Microneurography consists in measurement of sympathetic nerve activity in the skin or in skeletal muscle (MSNA) [83]. It is known that sympathetic outflows happen in bursts, so measuring sympathetic activity is based on detection of these bursts. Microneurography is an invasive method, needing a skilled technician to be performed [84].

The analysis can be made in three ways: measurement of the burst frequency (count of the number of bursts occurring/minute), measurement of burst incidence (number of bursts occurring per 100 heart beats) or measurement of the total neural activity (sum of bursts amplitude in 1 min) [31, 84, 85].

### Radiotracer-derived measurements

As heart and kidneys are out of reach for microneurography, radiotracer-derived measurements have been developed. This method is based on the measurement of noradrenaline outflow to the circulation. Tritiated I-norepinephrine is infused in the patient’s circulation, allowing to measure organ-specific noradrenaline spill over to plasma by isotope dilution [86, 87]. 123I-metaiodobenzylguanidine (123I-MIBG) is a norepinephrine analogue that can be used for non-invasive cardiac sympathetic neuronal activity assessment [88, 89].

### Other methods

SNS activity can also be measured by indirect methods such as heart rate variability, blood pressure variability or arterial baroreflex sensitivity.

#### Heart rate variability

Heart rate variability (HRV) is the variation in time of the heart rate around the mean value of the patient between two heart beats. A decrease in the HRV is seen during SNS hyperactivity and is correlated with an increased risk of cardiac mortality and is a predictor risk factor for cardiovascular events [40].

It can be measured through a few-minutes or 24-h ECG recordings that can accurately sense RR intervals, and calculated using several indexes: Time domain indexes, Frequency indexes or nonlinear indexes [90]. HRV is also used to assess cardiac vagal tone [89, 91].

#### Blood pressure variability

Variability in blood pressure is classified as short-term, mid-term and long-term variability and increases according to conditions depending on sympathetic activity. Mid- and long-term variability can be measured through ambulatory BP monitoring [92] while short-term variability can be measured by spectral analysis of blood pressure measured using finger plethysmographic devices [93].

#### Arterial baroreflex sensitivity

Baroreflex sensitivity determines the capability of the SNS to respond to changes in blood pressure sensed at the level of the carotid sinus and aortic arch [94]. It can be measured by application of a neck chamber (stimulation of carotid baroreflex), the use of vasoactive drugs (detection of systemic pressure changes), and the Valsalva maneuver (to detect bradycardic responses). To determine baroreflex sensitivity, physicians must analyse the relation between diastolic blood pressure and MSNA [95, 96].

The neck chambers measure baroreflex sensitivity stretching and compressing the baroreceptors via negative and positive pressures applied to the anterior neck [97]. Injection of a vasoactive drug (angiotensin or phenylephrine) allows a recording of ECG and beat-to-beat arterial pressure [98].

Multiple studies have analysed cross-correlation between HR and BP variabilities, which may account for changes in the autonomic nervous activity [99, 100].

## Effects of hypertension management strategies on the SNS

Recommendations for the treatment of hypertension include lifestyle changes (i.e., diet, weight loss, exercise) either alone or in combination with pharmacotherapies [1]. Reduction in sympathetic overdrive is expected to trigger a series of favourable cardiovascular and metabolic consequences [28], as shown with several of these hypertension management strategies.

### Lifestyle modifications

Lifestyle changes are the first line of treatment for hypertension [1]. While exercise reduces blood pressure and is protective against CVD, it does not appear to affect blood pressure, lipid levels or risk of diabetes [39]. A high-protein specialised eating plan, the Dietary Approach to Stop Hypertension (DASH), with an emphasis on fruit and vegetables, low-fat dairy, wholegrain cereals, vegetables and nuts, lean meats, poultry and fish, has been shown to reduce blood pressure and lipid levels, and improve metabolic markers when used as part of a weight-loss program [38]. Additionally, weight loss has been shown to depress SNS activity, with resultant improvements in insulin clearance, and reduced peripheral vascular resistance [38]. Central sympathetic outflow has also been implicated in the physiology of salt-sensitive blood pressure changes [101], and high sodium intake is associated with parasympathetic inhibition, dyslipidemia and inflammation in patients with mild hypertension [102].

In the CALERIE trial (Comprehensive Assessment of Long-term Effects of Reducing Intake of Energy) [62], patients were divided in three groups: calorie restriction (decrease of 25% of energy intake), calorie restriction and exercise (decrease of 12.5% associated with an increase of 12.5% in energy expenditure) or low-calorie diet until decrease of 15% of weight, followed by weight maintenance. After 6 months of trial, in all groups, the SNS activity was decreased whereas the PNS was increased but the changes reached significance only in the second group. Therefore, the results suggest that weight loss is an important factor to improve the SNS/PNS balance, especially when calorie restriction is combined with exercise.

### Catheter-based renal denervation

Catheter-based renal denervation has been explored as a treatment for drug-resistant hypertension [103, 104]. Renal denervation decreased blood pressure and reduced cardiovascular morbidity [105,106]. As well as the effects on blood pressure, renal denervation has demonstrated a decrease in muscle sympathetic nerve activity with a reduction of SNS-mediated effects such as improved glycaemic control and insulin resistance, and decreased total peripheral resistance [103, 104, 107]. Observed positive effects of renal denervation on the cardiovascular system include reduced arterial stiffness (lower pulse wave velocity), reduced left ventricular mass, improved heart failure symptoms and diastolic function, and increased left ventricular ejection fraction [103, 107]. Heart rate is also significantly reduced through renal denervation [108]. With regards to the effects on the kidney, renal denervation has been shown to reduce renal noradrenaline spill over and plasma renin release [109]. Interestingly, a recent paper showed that a baseline 24-h heart rate above the median (73.5 bpm), suggesting a higher sympathetic activity, predicted greater BP reductions after renal denervation and may allow physicians to select patients likely to respond to the procedure [108].

### Pharmacological strategies

Several pharmacological strategies can be set up to decrease blood pressure, targeting either RAAS (angiotensin converting enzyme inhibitors (ACEis)/angiotensin receptor blockers (ARBs)), cardiac output (β-blockers), peripheral vascular resistance (calcium channel blockers (CCB) or sodium reabsorption (diuretics).

Pharmacological therapies for the management of hypertension via inhibition of the SNS include beta-blockers and imidazoline receptor agonists, targeting beta-adrenergic and imidazoline I_1_ receptors, respectively [9, 10]. While ACEis and ARBs act by antagonising the renin–angiotensin–aldosterone system, they appear to have little or no effect on the SNS [110, 111].

#### Beta-blockers

Beta-blockers, also indicated for the treatment of heart failure [79, 112], decrease SNS activation by antagonizing beta-adrenergic receptors [10], and have potential beneficial effects on cardiac fibrosis. While they reduce SNS activation and cardiovascular outcomes in patients with heart failure and reduced LV ejection fraction, they also have unwanted negative metabolic effects including insulin resistance, dyslipidemia and reduced glycaemic control [10, 31, 113]. They also increase weight gain [2, 48].

#### Selective imidazoline receptor agonists.

**Fig. 3:**
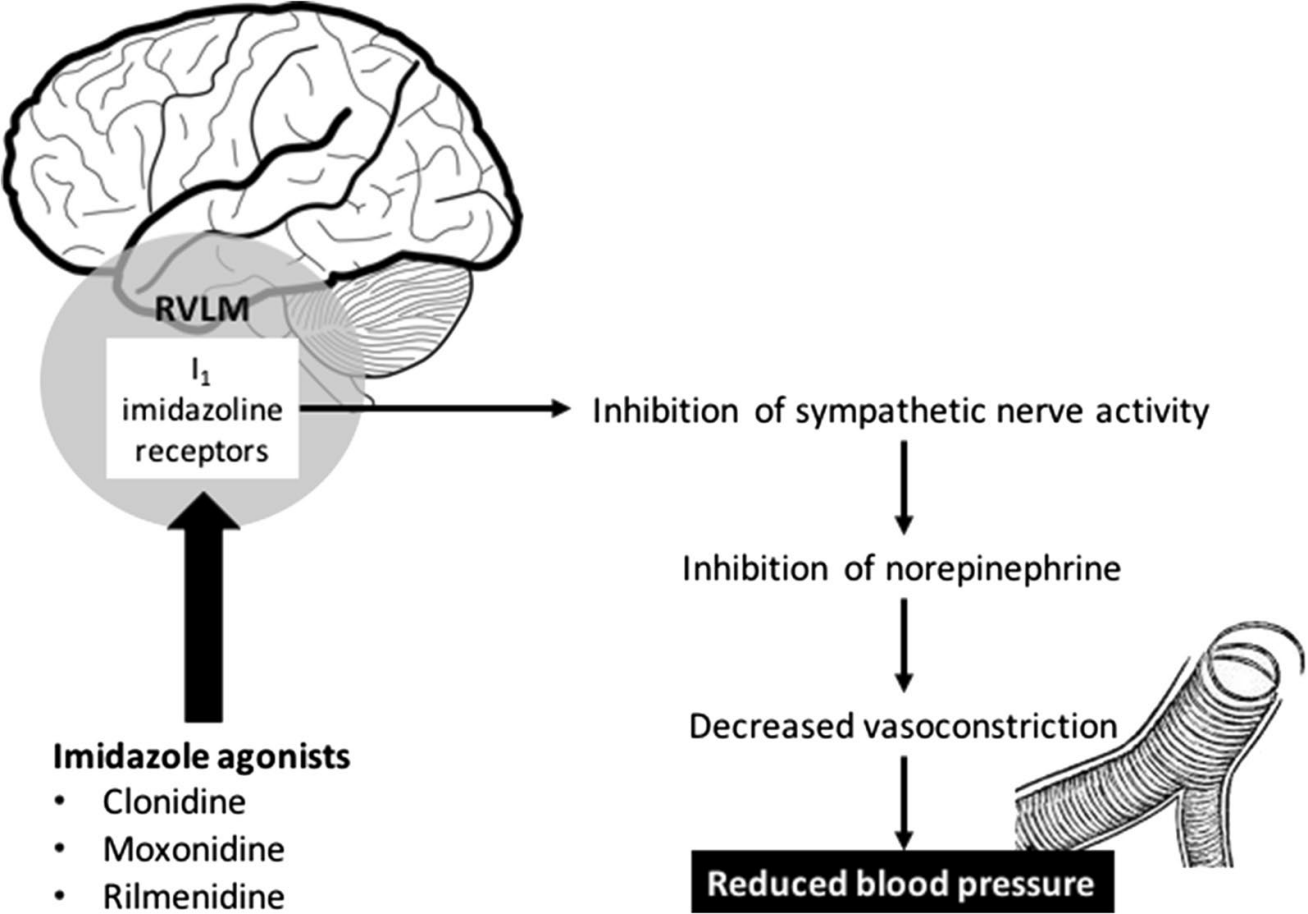
Mechanisms of action of imidazoline receptor agonists

**Fig. 4:**
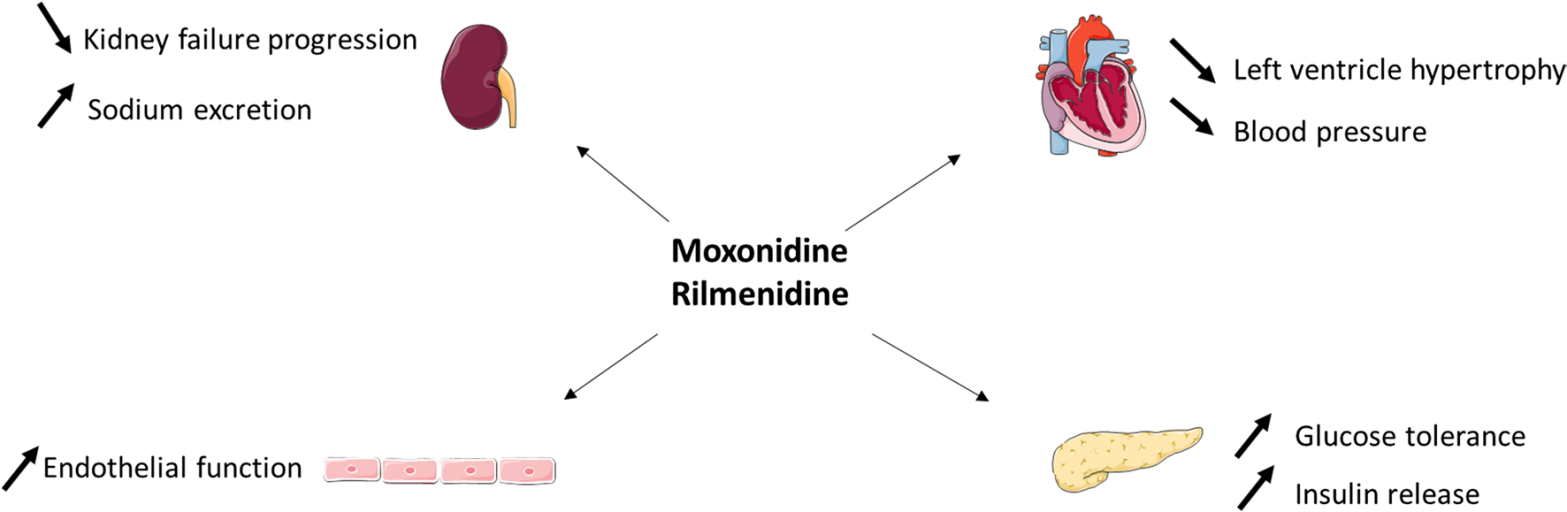
Metabolic and cardiovascular effects of selective imidazoline receptor agonists moxonidine and rilmenidine

Imidazoline receptor agonists (e.g., clonidine, moxonidine, rilmenidine) act on the imidazoline I_1_ receptor [9]. Concentrated within the rostral ventrolateral medulla (RVLM), a part of the brainstem involved in sympathetic control of blood pressure, imidazoline I_1_ receptors are important for the regulation of sympathetic drive [114, 115]. It is believed that the I_1_ receptor may belong to the neurocytokine receptor family, since its signalling pathways are similar to those of interleukins [114]. Of the imidazoline agonists, clonidine stimulates both α_2_ receptors and imidazoline I_1_ receptors, whereas moxonidine and rilmenidine are considered as selective imidazoline receptor agonists (SIRAs), activating only imidazoline I_1_ receptors within the RVLM [9, 116], avoiding thus side effects seen with clonidine (mainly tiredness, drowsiness and sedation). Increased neuronal activity in the RVLM by direct stimulation of I_1_ receptors by imidazoline agonists decreases sympathetic outflow, resulting in a fall in blood pressure (Fig. 3) [115]. I_1_ receptors are also localized on the plasma membrane of the neurons of the adrenal medulla, renal epithelium, pancreatic islets, platelets, and the prostate [114, 117]. In line with these findings, rilmenidine has demonstrated inhibition of cardiac baroreflex sympathetic activity, with subsequent reduction in heart rate [118], and beneficial effects of moxonidine include enhanced sodium excretion, improved insulin resistance (clamp study) [119] and glucose tolerance and protection against hypertensive target organ damage (e.g., kidney disease, cardiac hypertrophy) [120]. Moxonidine was also shown to decrease kidney failure progression, reduce left ventricular hypertrophy and to improve endothelial function (first step to atherosclerosis development) in hypertensive patients [116, 120]. In the MERSY study [121] administration of moxonidine daily (0.2–0.4 mg) for 6 months as monotherapy or in combination with another antihypertensive treatment in hypertensive patients with a metabolic syndrome was shown to improve blood pressure control, to reduce the body weight and to act on metabolic parameters as lipid fractions and fasting plasma glucose (Fig. 4). Recently, an international real-world medical research shown that in the surveyed countries, physicians considered SIRAs as a useful therapeutic option, frequently prescribed for hypertensive patients with metabolic outcomes [122].

## Conclusions

Hypertension is a multifactorial disease process that is a well-established cardiovascular risk factor. Overactivation of the SNS plays a central role in the aetiology of hypertension and has also been linked to several comorbidities commonly associated with hypertension, as metabolic syndrome, diabetes mellitus, dyslipidemia or sleep apnea syndrome. Some of the treatments for hypertension, particularly selective imidazoline receptor agonists, target the SNS and have demonstrated beneficial cardiovascular, renal, and metabolic effects in addition to lowering blood pressure.

## Data Availability

Not applicable.

## Abbreviations

SNS: Sympathetic nervous system
CVD: Cardiovascular diseases
ANS: Autonomic nervous system
RAAS: Renin–angiotensin–aldosterone system
PNS: Parasympathetic nervous system
GLP-1: Glucagon-like peptide 1
MSNA: Muscle sympathetic nerve activity
DASH: Dietary Approach to Stop Hypertension
CALERIE: Comprehensive Assessment of Long-term Effects of Reducing Intake of Energy
ACEis: Angiotensin converting enzyme inhibitors
ARBs: Angiotensin receptor blockers
CCB: Calcium channel blockers
RVLM: Rostral ventrolateral medulla
SIRAs: Selective imidazoline receptor agonists

## References

[1] Whelton PK, Carey RM, Aronow WS, Casey DE, Collins KJ, Dennison Himmelfarb C (2018). 2017 ACC/AHA/AAPA/ABC/ACPM/AGS/APhA/ASH/ASPC/NMA/PCNA guideline for the prevention, detection, evaluation, and management of high blood pressure in adults: executive summary: a report of the American College of Cardiology/American Heart Association Task Force on Clinical Practice Guidelines. Circulation.

[2] Williams B, Mancia G, Spiering W, Agabiti Rosei E, Azizi M, Burnier M (2018). 2018 ESC/ESH guidelines for the management of arterial hypertension. Eur Heart J.

[3] Coats AJS, Cruickshank JM (2014). Hypertensive subjects with type-2 diabetes, the sympathetic nervous system, and treatment implications. Int J Cardiol.

[4] DeMarco VG, Aroor AR, Sowers JR (2014). The pathophysiology of hypertension in patients with obesity. Nat Rev Endocrinol.

[5] Mills KT, Bundy JD, Kelly TN, Reed JE, Kearney PM, Reynolds K (2016). Global disparities of hypertension prevalence and control: a systematic analysis of population-based studies from 90 countries. Circulation.

[6] NCD Risk Factor Collaboration (NCD-RisC) (2017). Worldwide trends in blood pressure from 1975 to 2015: a pooled analysis of 1479 population-based measurement studies with 19·1 million participants. Lancet Lond Engl.

[7] Esler M (2000). The sympathetic system and hypertension. Am J Hypertens.

[8] Mancia G, Grassi G (2014). The autonomic nervous system and hypertension. Circ Res.

[9] Sica DA (2007). Centrally acting antihypertensive agents: an update. J Clin Hypertens Greenwich Conn.

[10] Fonseca VA (2010). Effects of beta-blockers on glucose and lipid metabolism. Curr Med Res Opin.

[11] Savica V, Bellinghieri G, Kopple JD (2010). The effect of nutrition on blood pressure. Annu Rev Nutr.

[12] Chan Q, Stamler J, Griep LMO, Daviglus ML, Horn LV, Elliott P (2016). An update on nutrients and blood pressure. J Atheroscler Thromb.

[13] Hall JE, Granger JP, do Carmo JM, da Silva AA, Dubinion J, George E (2012). Hypertension: physiology and pathophysiology. Compr Physiol.

[14] Te Riet L, van Esch JHM, Roks AJM, van den Meiracker AH, Danser AHJ (2015). Hypertension: renin-angiotensin-aldosterone system alterations. Circ Res.

[15] Patel S, Rauf A, Khan H, Abu-Izneid T (2017). Renin-angiotensin-aldosterone (RAAS): the ubiquitous system for homeostasis and pathologies. Biomed Pharmacother.

[16] Hartupee J, Mann DL (2017). Neurohormonal activation in heart failure with reduced ejection fraction. Nat Rev Cardiol.

[17] Miller AJ, Arnold AC (2019). The renin-angiotensin system in cardiovascular autonomic control: recent developments and clinical implications. Clin Auton Res.

[18] Grassi G, Ram VS (2016). Evidence for a critical role of the sympathetic nervous system in hypertension. J Am Soc Hypertens JASH.

[19] Wehrwein EA, Orer HS, Barman SM (2016). Overview of the anatomy, physiology, and pharmacology of the autonomic nervous system. Compr Physiol.

[20] 20.4 Homeostatic Regulation of the Vascular System—Douglas College Human Anatomy and Physiology I, 1st edition. https://pressbooks.bccampus.ca/dcbiol11031109/chapter/20-4-homeostatic-regulation-of-the-vascular-system/. Accessed 30 Sept 2019.

[21] Rui L (2014). Energy metabolism in the liver. Compr Physiol.

[22] Ahrén B (2000). Autonomic regulation of islet hormone secretion—implications for health and disease. Diabetologia.

[23] Pénicaud L (2017). Autonomic nervous system and pancreatic islet blood flow. Biochimie.

[24] Yahagi N (2017). Hepatic control of energy metabolism via the autonomic nervous system. J Atheroscler Thromb.

[25] Grassi G (1979). Assessment of sympathetic cardiovascular drive in human hypertension: achievements and perspectives. Hypertens Dallas Tex.

[26] Cosson E, Valensi P, Laude D, Mesangeau D, Dabire H (2009). Arterial stiffness and the autonomic nervous system during the development of Zucker diabetic fatty rats. Diabetes Metab.

[27] Cosson E, Herisse M, Laude D, Thomas F, Valensi P, Attali J-R (2007). Aortic stiffness and pulse pressure amplification in Wistar-Kyoto and spontaneously hypertensive rats. Am J Physiol Heart Circ Physiol.

[28] Grassi G, Mark A, Esler M (2015). The sympathetic nervous system alterations in human hypertension. Circ Res.

[29] Manolis AJ, Poulimenos LE, Kallistratos MS, Gavras I, Gavras H (2014). Sympathetic overactivity in hypertension and cardiovascular disease. Curr Vasc Pharmacol.

[30] Preckel D, von Känel R (2004). Regulation of hemostasis by the sympathetic nervous system: any contribution to coronary artery disease?. Hear Excell Cardiovasc Trials.

[31] Parati G, Esler M (2012). The human sympathetic nervous system: its relevance in hypertension and heart failure. Eur Heart J.

[32] Triposkiadis F, Karayannis G, Giamouzis G, Skoularigis J, Louridas G, Butler J (2009). The sympathetic nervous system in heart failure physiology, pathophysiology, and clinical implications. J Am Coll Cardiol.

[33] Manzella D, Barbieri M, Rizzo MR, Ragno E, Passariello N, Gambardella A (2001). Role of free fatty acids on cardiac autonomic nervous system in noninsulin-dependent diabetic patients: effects of metabolic control. J Clin Endocrinol Metab.

[34] Carnagarin R, Matthews VB, Herat LY, Ho JK, Schlaich MP (2018). Autonomic regulation of glucose homeostasis: a specific role for sympathetic nervous system activation. Curr Diabetes Rep.

[35] Brook RD, Julius S (2000). Autonomic imbalance, hypertension, and cardiovascular risk. Am J Hypertens.

[36] Morton GJ, Muta K, Kaiyala KJ, Rojas JM, Scarlett JM, Matsen ME (2017). Evidence that the sympathetic nervous system elicits rapid, coordinated, and reciprocal adjustments of insulin secretion and insulin sensitivity during cold exposure. Diabetes.

[37] Schumann U, Jenkinson CP, Alt A, Zügel M, Steinacker JM, Flechtner-Mors M (2017). Sympathetic nervous system activity and anti-lipolytic response to iv-glucose load in subcutaneous adipose tissue of obese and obese type 2 diabetic subjects. PLoS ONE.

[38] Straznicky NE, Grima MT, Sari CI, Lambert EA, Phillips SE, Eikelis N (2015). Reduction in peripheral vascular resistance predicts improvement in insulin clearance following weight loss. Cardiovasc Diabetol.

[39] Edwards KM, Wilson KL, Sadja J, Ziegler MG, Mills PJ (2011). Effects on blood pressure and autonomic nervous system function of a 12-week exercise or exercise plus DASH-diet intervention in individuals with elevated blood pressure. Acta Physiol Oxf Engl.

[40] Goit RK, Ansari AH (2016). Reduced parasympathetic tone in newly diagnosed essential hypertension. Indian Heart J.

[41] Diabetes in Cardiovascular Disease: a companion to Braunwald’s Heart Disease. 1st edition. https://www.elsevier.com/books/diabetes-in-cardiovascular-disease-a-companion-to-braunwalds-heart-disease/mcguire/978-1-4557-5418-2. Accessed 3 Oct 2019.

[42] Ayad F, Belhadj M, Pariés J, Attali JR, Valensi P (2010). Association between cardiac autonomic neuropathy and hypertension and its potential influence on diabetic complications. Diabet Med J Br Diabet Assoc.

[43] Thorp AA, Schlaich MP (2015). Relevance of sympathetic nervous system activation in obesity and metabolic syndrome. J Diabetes Res.

[44] Chapelot D, Charlot K (2019). Physiology of energy homeostasis: models, actors, challenges and the glucoadipostatic loop. Metabolism.

[45] Grassi G, Seravalle G, Dell’Oro R, Turri C, Bolla GB, Mancia G (2000). Adrenergic and reflex abnormalities in obesity-related hypertension. Hypertension.

[46] Lindmark S, Lönn L, Wiklund U, Tufvesson M, Olsson T, Eriksson JW (2005). Dysregulation of the autonomic nervous system can be a link between visceral adiposity and insulin resistance. Obes Res.

[47] Guarino D, Nannipieri M, Iervasi G, Taddei S, Bruno RM (2017). The role of the autonomic nervous system in the pathophysiology of obesity. Front Physiol.

[48] Carnagarin R, Matthews V, Gregory C, Schlaich MP (2018). Pharmacotherapeutic strategies for treating hypertension in patients with obesity. Expert Opin Pharmacother.

[49] Tentolouris N, Tsigos C, Perea D, Koukou E, Kyriaki D, Kitsou E (2003). Differential effects of high-fat and high-carbohydrate isoenergetic meals on cardiac autonomic nervous system activity in lean and obese women. Metabolism.

[50] Nagai N, Sakane N, Hamada T, Kimura T, Moritani T (2005). The effect of a high-carbohydrate meal on postprandial thermogenesis and sympathetic nervous system activity in boys with a recent onset of obesity. Metabolism.

[51] Straznicky NE, Lambert GW, Masuo K, Dawood T, Eikelis N, Nestel PJ (2009). Blunted sympathetic neural response to oral glucose in obese subjects with the insulin-resistant metabolic syndrome. Am J Clin Nutr.

[52] Valensi P, Ngoc PB, Idriss S, Paries J, Cazes P, Lormeau B (1999). Haemodynamic response to an isometric exercise test in obese patients: Influence of autonomic dysfunction. Int J Obes Relat Metab Disord.

[53] Valensi P, Smagghue O, Pariès J, Velayoudon P, Lormeau B, Attali JR (2000). Impairment of skin vasoconstrictive response to sympathetic activation in obese patients: Influence of rheological disorders. Metabolism.

[54] Ma D, Feitosa MF, Wilk JB, Laramie JM, Yu K, Leiendecker-Foster C (1979). Leptin is associated with blood pressure and hypertension in women from the National Heart, Lung, and Blood Institute Family Heart Study. Hypertens Dallas Tex.

[55] Bell BB, Rahmouni K (2016). Leptin as a mediator of obesity-induced hypertension. Curr Obes Rep.

[56] Vaz M, Jennings G, Turner A, Cox H, Lambert G, Esler M (1997). Regional sympathetic nervous activity and oxygen consumption in obese normotensive human subjects. Circulation.

[57] Lee ZS, Critchley JA, Tomlinson B, Young RP, Thomas GN, Cockram CS (2001). Urinary epinephrine and norepinephrine interrelations with obesity, insulin, and the metabolic syndrome in Hong Kong Chinese. Metabolism.

[58] Grassi G, Seravalle G, Cattaneo BM, Bolla GB, Lanfranchi A, Colombo M (1995). Sympathetic activation in obese normotensive subjects. Hypertens Dallas Tex 1979.

[59] Grassi G, Dell’Oro R, Facchini A, Quarti Trevano F, Bolla GB, Mancia G (2004). Effect of central and peripheral body fat distribution on sympathetic and baroreflex function in obese normotensives. J Hypertens.

[60] Straznicky NE, Lambert EA, Lambert GW, Masuo K, Esler MD, Nestel PJ (2005). Effects of dietary weight loss on sympathetic activity and cardiac risk factors associated with the metabolic syndrome. J Clin Endocrinol Metab.

[61] Lambert EA, Esler MD, Schlaich MP, Dixon J, Eikelis N, Lambert GW (1979). Obesity-associated organ damage and sympathetic nervous activity. Hypertens Dallas Tex 1979.

[62] de Jonge L, Moreira EAM, Martin CK, Ravussin E, Pennington CALERIE Team (2010). Impact of 6-month caloric restriction on autonomic nervous system activity in healthy, overweight, individuals. Obes Silver Spring Md.

[63] Van De Borne P, Hausberg M, Hoffman RP, Mark AL, Anderson EA (1999). Hyperinsulinemia produces cardiac vagal withdrawal and nonuniform sympathetic activation in normal subjects. Am J Physiol-Regul Integr Comp Physiol.

[64] Vollenweider P, Tappy L, Randin D, Schneiter P, Jéquier E, Nicod P (1993). Differential effects of hyperinsulinemia and carbohydrate metabolism on sympathetic nerve activity and muscle blood flow in humans. J Clin Invest.

[65] Hall JE, do Carmo JM, da Silva AA, Wang Z, Hall ME (2015). Obesity-induced hypertension: interaction of neurohumoral and renal mechanisms. Circ Res.

[66] Chiheb S, Cosson E, Banu I, Hamo-Tchatchouang E, Cussac-Pillegand C, Nguyen M (2016). Are obese individuals with no feature of metabolic syndrome but increased waist circumference really healthy? A cross sectional study. Exp Clin Endocrinol Diabetes.

[67] Valensi P, Doaré L, Perret G, Germack R, Pariès J, Mesangeau D (2003). Cardiovascular vagosympathetic activity in rats with ventromedial hypothalamic obesity. Obes Res.

[68] Valensi P, Chiheb S, Fysekidis M (2013). Insulin- and glucagon-like peptide-1-induced changes in heart rate and vagosympathetic activity: why they matter. Diabetologia.

[69] Baggio LL, Ussher JR, McLean BA, Cao X, Kabir MG, Mulvihill EE (2017). The autonomic nervous system and cardiac GLP-1 receptors control heart rate in mice. Mol Metab.

[70] Rowlands J, Heng J, Newsholme P, Carlessi R (2018). Pleiotropic effects of GLP-1 and analogs on cell signaling, metabolism, and function. Front Endocrinol.

[71] González-García I, Milbank E, Diéguez C, López M, Contreras C (2019). Glucagon, GLP-1 and thermogenesis. Int J Mol Sci.

[72] Barragán JM, Rodríguez RE, Blázquez E (1994). Changes in arterial blood pressure and heart rate induced by glucagon-like peptide-1-(7–36) amide in rats. Am J Physiol.

[73] Barragán JM, Rodríguez RE, Eng J, Blázquez E (1996). Interactions of exendin-(9–39) with the effects of glucagon-like peptide-1-(7–36) amide and of exendin-4 on arterial blood pressure and heart rate in rats. Regul Pept.

[74] Yamamoto H, Kishi T, Lee CE, Choi BJ, Fang H, Hollenberg AN (2003). Glucagon-like peptide-1-responsive catecholamine neurons in the area postrema link peripheral glucagon-like peptide-1 with central autonomic control sites. J Neurosci.

[75] Yamamoto H, Lee CE, Marcus JN, Williams TD, Overton JM, Lopez ME (2002). Glucagon-like peptide-1 receptor stimulation increases blood pressure and heart rate and activates autonomic regulatory neurons. J Clin Invest.

[76] Griffioen KJ, Wan R, Okun E, Wang X, Lovett-Barr MR, Li Y (2011). GLP-1 receptor stimulation depresses heart rate variability and inhibits neurotransmission to cardiac vagal neurons. Cardiovasc Res.

[77] Nyström T, Gutniak MK, Zhang Q, Zhang F, Holst JJ, Ahrén B (2004). Effects of glucagon-like peptide-1 on endothelial function in type 2 diabetes patients with stable coronary artery disease. Am J Physiol-Endocrinol Metab.

[78] Sforza E, Martin MS, Barthélémy JC, Roche F (2016). Is there an association between altered baroreceptor sensitivity and obstructive sleep apnoea in the healthy elderly?. ERJ Open Res.

[79] Zhang DY, Anderson AS (2014). The sympathetic nervous system and heart failure. Cardiol Clin.

[80] Somers VK, Dyken ME, Clary MP, Abboud FM (1995). Sympathetic neural mechanisms in obstructive sleep apnea. J Clin Invest.

[81] Carnagarin R, Lambert GW, Kiuchi MG, Nolde JM, Matthews VB, Eikelis N (2019). Effects of sympathetic modulation in metabolic disease. Ann N Y Acad Sci.

[82] Borel A-L, Tamisier R, Böhme P, Priou P, Avignon A, Benhamou P-Y (2019). Obstructive sleep apnoea syndrome in patients living with diabetes: which patients should be screened?. Diabetes Metab.

[83] White DW, Shoemaker JK, Raven PB (2015). Methods and considerations for the analysis and standardization of assessing muscle sympathetic nerve activity in humans. Auton Neurosci.

[84] Macefield V (2013). Sympathetic microneurography. Handb Clin Neurol.

[85] Greaney J, Kenney W (2017). Measuring and quantifying skin sympathetic nervous system activity in humans. J Neurophysiol.

[86] Mathias CJ, Bannister SR (2013). Autonomic failure: a textbook of clinical disorders of the autonomic nervous system.

[87] Esler M, Leonard P, O’Dea K, Jackman G, Jennings G, Korner P (1982). Biochemical quantification of sympathetic nervous activity in humans using radiotracer methodology: fallibility of plasma noradrenaline measurements. J Cardiovasc Pharmacol.

[88] Verberne HJ, Brewster LM, Somsen GA, van Eck-Smit BLF (2008). Prognostic value of myocardial 123I-metaiodobenzylguanidine (MIBG) parameters in patients with heart failure: a systematic review. Eur Heart J.

[89] Bernardi L, Spallone V, Stevens M, Hilsted J, Frontoni S, Pop-Busui R (2011). Methods of investigation for cardiac autonomic dysfunction in human research studies: investigation methods for cardiac autonomic function. Diabetes Metab Res Rev.

[90] Buccelletti E, Gilardi E, Scaini E, Galiuto L, Persiani R, Biondi A (2009). Heart rate variability and myocardial infarction: systematic literature review and metanalysis. Eur Rev Med Pharmacol Sci.

[91] Ernst G (2017). Heart-rate variability—more than heart beats?. Front Public Health.

[92] Parati G (2005). Blood pressure variability: its measurement and significance in hypertension. J Hypertens Suppl.

[93] Constant I, Laude D, Elghozi JL, Murat I (1999). Assessment of short-term blood pressure variability in anesthetized children: a comparative study between intraarterial and finger blood pressure. J Clin Monit Comput.

[94] Rudas L, Crossman AA, Morillo CA, Halliwill JR, Tahvanainen KU, Kuusela TA (1999). Human sympathetic and vagal baroreflex responses to sequential nitroprusside and phenylephrine. Am J Physiol.

[95] Dutoit AP, Hart EC, Charkoudian N, Wallin BG, Curry TB, Joyner MJ (2010). Cardiac baroreflex sensitivity is not correlated to sympathetic baroreflex sensitivity within healthy, young humans. Hypertens Dallas Tex 1979.

[96] Laude D, Elghozi J-L, Girard A, Bellard E, Bouhaddi M, Castiglioni P (2004). Comparison of various techniques used to estimate spontaneous baroreflex sensitivity (the EuroBaVar study). Am J Physiol Regul Integr Comp Physiol.

[97] Eckberg DL (1980). Nonlinearities of the human carotid baroreceptor-cardiac reflex. Circ Res.

[98] La Rovere MT, Pinna GD, Raczak G (2008). Baroreflex sensitivity: measurement and clinical implications. Ann Noninvasive Electrocardiol.

[99] Yadav RL, Yadav PK, Yadav LK, Agrawal K, Sah SK, Islam MN (2017). Association between obesity and heart rate variability indices: an intuition toward cardiac autonomic alteration—a risk of CVD. Diabetes Metab Syndr Obes Targets Ther.

[100] Sugita N, Yoshizawa M, Abe M, Tanaka A, Chiba S, Yambe T, et al. Comparison of maximum cross-correlation coefficient between blood pressure and heart rate with traditional index associated with baroreflex sensitivity. In: 2008 30th annual international conference of the IEEE engineering in medicine and biology society. Vancouver, BC: IEEE; 2008. pp. 2574–7. 10.1109/IEMBS.2008.4649726 19163229

[101] Farquhar WB, Edwards DG, Jurkovitz CT, Weintraub WS (2015). Dietary sodium and health: more than just blood pressure. J Am Coll Cardiol.

[102] González SA, Forcada P, de Cavanagh EMV, Inserra F, Svane JC, Obregón S (2012). Sodium intake is associated with parasympathetic tone and metabolic parameters in mild hypertension. Am J Hypertens.

[103] Sánchez-Álvarez C, González-Vélez M, Stilp E, Ward C, Mena-Hurtado C (2014). Renal sympathetic denervation in the treatment of resistant hypertension. Yale J Biol Med.

[104] Osborn JW, Banek CT (2018). Catheter-based renal nerve ablation as a novel hypertension therapy: lost, and then found in translation. Hypertens Dallas Tex 1979.

[105] Azizi M, Sapoval M, Gosse P, Monge M, Bobrie G, Delsart P (2015). Optimum and stepped care standardised antihypertensive treatment with or without renal denervation for resistant hypertension (DENERHTN): a multicentre, open-label, randomised controlled trial. Lancet Lond Engl.

[106] Persu A, Maes F, Renkin J, Pathak A (2020). Renal denervation in hypertensive patients: back to anatomy?. Hypertension.

[107] Hatipoglu E, Ferro A (2013). Catheter-based renal denervation for treatment of resistant hypertension. JRSM Cardiovasc Dis.

[108] Böhm M, Mahfoud F, Townsend RR, Kandzari DE, Pocock S, Ukena C (2019). Ambulatory heart rate reduction after catheter-based renal denervation in hypertensive patients not receiving anti-hypertensive medications: data from SPYRAL HTN-OFF MED, a randomized, sham-controlled, proof-of-concept trial. Eur Heart J.

[109] Kannan A, Medina RI, Nagajothi N, Balamuthusamy S (2014). Renal sympathetic nervous system and the effects of denervation on renal arteries. World J Cardiol.

[110] Johansson M, Elam M, Rundqvist B, Eisenhofer G, Herlitz H, Jensen G (2000). Differentiated response of the sympathetic nervous system to angiotensin-converting enzyme inhibition in hypertension. Hypertens Dallas Tex 1979.

[111] Krum H, Lambert E, Windebank E, Campbell DJ, Esler M (2006). Effect of angiotensin II receptor blockade on autonomic nervous system function in patients with essential hypertension. Am J Physiol Heart Circ Physiol.

[112] Chatterjee S, Biondi-Zoccai G, Abbate A, D’Ascenzo F, Castagno D, Van Tassell B (2013). Benefits of β blockers in patients with heart failure and reduced ejection fraction: network meta-analysis. BMJ.

[113] Del Colle S, Morello F, Rabbia F, Milan A, Naso D, Puglisi E (2007). Antihypertensive drugs and the sympathetic nervous system. J Cardiovasc Pharmacol.

[114] Ernsberger P (1999). The I1-imidazoline receptor and its cellular signaling pathways. Ann N Y Acad Sci.

[115] Waller, Sampson. Medical pharmacology and therapeutics by Derek G. Waller and Anthony P. Sampson|eBook on Inkling. 5th edition. 2018. https://www.inkling.com/store/book/waller-medical-pharmacology-therapeutics-5e/. Accessed 30 Sept 2019.

[116] Edwards LP, Brown-Bryan TA, McLean L, Ernsberger P (2012). Pharmacological properties of the central antihypertensive agent, moxonidine. Cardiovasc Ther.

[117] Ernsberger P, Graves ME, Graff LM, Zakieh N, Nguyen P, Collins LA (1995). I1-imidazoline receptors. Definition, characterization, distribution, and transmembrane signaling. Ann N Y Acad Sci.

[118] Head GA, Burke SL (2000). I1 imidazoline receptors in cardiovascular regulation: the place of rilmenidine. Am J Hypertens.

[119] Haenni A, Lithell H (1999). Moxonidine improves insulin sensitivity in insulin-resistant hypertensives. J Hypertens Suppl.

[120] Fenton C, Keating GM, Lyseng-Williamson KA (2006). Moxonidine: a review of its use in essential hypertension. Drugs.

[121] Chazova I, Schlaich MP (2013). Improved hypertension control with the imidazoline agonist moxonidine in a multinational metabolic syndrome population: principal results of the MERSY Study. Int J Hypertens.

[122] Schlaich MP, Almahmeed W, Arnaout S, Prabhakaran D, Zhernakova J, Zvartau N (2020). The role of selective imidazoline receptor agonists in modern hypertension management: an international real-world survey (STRAIGHT). Curr Med Res Opin.

